# Impact of Renal Function on Long-Term Clinical Outcomes in Patients With Coronary Chronic Total Occlusions: Results From an Observational Single-Center Cohort Study During the Last 12 Years

**DOI:** 10.3389/fcvm.2020.550428

**Published:** 2020-11-16

**Authors:** Lei Guo, Huaiyu Ding, Haichen Lv, Xiaoyan Zhang, Lei Zhong, Jian Wu, Jiaying Xu, Xuchen Zhou, Rongchong Huang

**Affiliations:** ^1^Department of Cardiology, The First Affiliated Hospital of Dalian Medical University, Dalian, China; ^2^Department of Radiology, Fuyang Hospital of Anhui Medical University, Fuyang, China; ^3^Department of Cardiology, Capital Medical University Affiliated Beijing Friendship Hospital, Beijing, China

**Keywords:** chronic total occlusions, medical therapy, outcomes, percutaneous coronary intervention, renal function

## Abstract

**Background:** The number of coronary chronic total occlusion (CTO) patients with renal insufficiency is huge, and limited data are available on the impact of renal insufficiency on long-term clinical outcomes in CTO patients. We aimed to investigate clinical outcomes of CTO percutaneous coronary intervention (PCI) vs. medical therapy (MT) in CTO patients according to baseline renal function.

**Methods:** In the study population of 2,497, 1,220 patients underwent CTO PCI and 1,277 patients received MT. Patients were divided into four groups based on renal function: group 1 [estimated glomerular filtration rate (eGFR) ≥ 90 ml/min/1.73 m^2^], group 2 (60 ≤ eGFR <90 ml/min/1.73 m^2^), group 3 (30 ≤ eGFR <60 ml/min/1.73 m^2^), and group 4 (eGFR <30 ml/min/1.73 m^2^). Major adverse cardiac event (MACE) was the primary end point.

**Results:** Median follow-up was 2.6 years. With the decline in renal function, MACE (*p* < 0.001) and cardiac death (*p* < 0.001) were increased. In group 1 and group 2, MACE occurred less frequently in patients with CTO PCI, as compared to patients in the MT group (15.6% vs. 22.8%, *p* < 0.001; 15.6% vs. 26.5%, *p* < 0.001; respectively). However, there was no significant difference in terms of MACE between CTO PCI and MT in group 3 (21.1% vs. 28.7%, *p* = 0.211) and group 4 (28.6% vs. 50.0%, *p* = 0.289). MACE was significantly reduced for patients who received successful CTO PCI compared to patients with MT (16.7% vs. 22.8%, *p* = 0.006; 16.3% vs. 26.5%, *p* = 0.003, respectively) in group 1 and group 2. eGFR < 30 ml/min/1.73 m^2^, age, male gender, diabetes mellitus, heart failure, multivessel disease, and MT were identified as independent predictors for MACE in patients with CTOs.

**Conclusions:** Renal impairment is associated with MACE in patients with CTOs. For treatment of CTO, compared with MT alone, CTO PCI may reduce the risk of MACE in patients without chronic kidney disease (CKD). However, reduced MACE from CTO PCI among patients with CKD was not observed. Similar beneficial effects were observed in patients without CKD who underwent successful CTO procedures.

## Introduction

Renal insufficiency is associated with an increased risk of mortality and cardiovascular events in patients with coronary artery disease (CAD) after percutaneous coronary intervention (PCI) or surgical revascularization (1–3). Coronary chronic total occlusions (CTOs), as a special type of CAD, are observed in 10–30% of all diagnostic coronary angiography and remain a challenging obstacle in coronary intervention (4–6). The number of CTO patients with renal insufficiency was huge (7, 8). Renal insufficiency was identified as an important high-risk factor for adverse outcomes or death in patients with CTOs (9, 10). Successful CTO procedures have been reported to improve left ventricular function and reduce adverse clinical events (11–13). However, data on the impact of renal insufficiency on clinical outcomes in CTO patients are scarce. Further, the optimal treatment strategy for these high-risk subjects is unknown.

Therefore, this study aimed to investigate clinical outcomes of CTO PCI vs. medical therapy (MT) in CTO patients according to baseline renal function.

## Materials and Methods

### Study Population

From a total of 27,231 patients undergoing coronary angiography at our institution between January 2007 and December 2018, we identified 2,980 (10.9%) patients who had at least one CTO lesion. Patients who underwent coronary artery bypass grafting (CABG) and those with acute ST-segment elevation myocardial infarction (MI) within the preceding 2 days, history of CABG, missing renal function data, cardiogenic shock, or malignant tumor were excluded from the study (13), leaving a final population of 2,497 patients, treated either by PCI or by MT. Patients referred for revascularization had presence of angina symptoms, or myocardial viability or inducible ischemia based on non-invasive imaging, including dimensional echocardiography, cardiac magnetic resonance imaging, or myocardial perfusion scintigraphy (14, 15). Baseline demographics and angiographic and procedural characteristics were collected and recorded. Patients were followed by telephone contacts or outpatient visits. The present study was approved by the review board of our institution and complied with the principles laid down in the Declaration of Helsinki.

### Definitions and End Points

Coronary CTO was defined as a true total occlusion with Thrombolysis In Myocardial Infarction (TIMI) 0 flow for >3 months (16). Duration was estimated according to clinical history or prior angiogram. Renal function was assessed according to the estimated glomerular filtration rate (eGFR) based on the Chronic Kidney Disease Epidemiology Collaboration (CKD-EPI) equation (17). Patients were divided into four groups according to baseline eGFR: group 1 (eGFR ≥ 90 ml/min/1.73 m^2^), group 2 (60 ≤ eGFR <90 ml/min/1.73 m^2^), group 3 (30 ≤ eGFR <60 ml/min/1.73 m^2^), and group 4 (eGFR <30 ml/min/1.73 m^2^). Chronic kidney disease (CKD) was defined as eGFR <60 ml/min/1.73 m^2^. Angiographic success of the CTO PCI was defined as <30% residual stenosis with TIMI grade ≥ 3 flow and no occlusion of a significant side branch, flow-limiting dissection, distal embolization, or angiographic thrombus (18). The primary end point for this study was major adverse cardiac event (MACE), which was defined as a composite of cardiac mortality, MI, or target-vessel revascularization (TVR). The secondary outcome was cardiac death. Definitions of cardiac death, MI, or TVR were described previously according to Standardized Definitions (8).

### Medical and Invasive Treatment

MT included statins, antiplatelet medication, β-blockers, nitrate, and renin–angiotensin system blockade. PCIs were performed in a standard manner. The patients received aspirin (100 mg) and clopidogrel (75 mg) daily before the intervention. After PCI, dual-antiplatelet medication was prescribed for at least 12 months. Patients with CKD received hydration to prevent the occurrence of acute kidney injury before and after the CTO procedure.

### Statistical Analysis

Data are presented as mean ± SD or as percentages. Kruskal–Wallis test was performed for comparison of continuous data, and chi-square test analysis or Fisher's exact test was used for categorical variables comparison. Long-term outcomes were determined by using Kaplan–Meier survival curves and compared by the log-rank test. A multivariate Cox regression model was used to identify the independent predictors of MACE at follow-up. Covariates that were with *p* < 0.05 on univariate analysis were considered candidate variables including age (per year increment), gender, heart failure, diabetes mellitus, left ventricular ejection fraction (LVEF) ≤ 40%, right coronary artery (RCA) CTO, multivessel disease, and MT. All statistical tests were two-sided and considered significant at *p* < 0.05. All analyses were conducted using IBM SPSS Statistics v24.0 (Chicago, IL) and Stata v15.1 (StataCorp LLC, TX).

## Results

### Baseline Characteristics

The prevalence of CTO was 10.9% in the present study. After exclusion, in the study population of 2,497, 1,220 patients underwent CTO PCI (710 patients had successful PCI and 510 patients had a failed PCI, and the success rate was 58.2%) and 1,277 patients received MT. Among this population, 1,391 (55.7%), 862 (34.5%), 212 (8.5%), and 32 (1.3%) patients were classified into groups 1–4, respectively. There were five patients with eGFR <15 ml/min/1.73 m^2^. The baseline clinical and angiographic characteristics are listed in Table 1. With decreasing renal function, patients tended to be older and had higher percentage of female, hypertension, and heart failure, but had a lower prevalence of smoker and lower LVEF. In angiographic characteristics, calcification, multivessel disease, and high Japanese-chronic total occlusion (J-CTO) score were more prevalent in patients with renal impairment. However, dyslipidemia, previous MI, familial history of CAD, the usage of medication except for aspirin, 2 CTO lesions, and LAD CTO were similar between the four groups. Procedural details and in-hospital death are present in Table 2.

**Table 1:** Baseline clinical and angiographic characteristics of all patients stratified by eGFR.

**Variables**	**All patients**	**Group 1 (eGFR ≥ 90 ml/min/1.73 m^**2**^)**	**Group 2 (60 ≤ eGFR <90 ml/min/1.73 m^**2**^)**	**Group 3 (30 ≤ eGFR <60 ml/min/1.73 m^**2**^)**	**Group 4 (eGFR <30 ml/min/1.73 m^**2**^)**	***P* value**
	***n* = 2,497**	***n* = 1,391**	***n* = 862**	***n* = 212**	***n* = 32**	
Age, years	64.2 ± 10.2	60.5 ± 9.5	68.2 ± 8.9	71.2 ± 9.2	70.9 ± 11.1	<0.001
Male	1,941 (77.7)	1,157 (83.2)	622 (72.2)	142 (67.0)	18 (56.3)	<0.001
Smoking	1,070 (42.8)	664 (47.7)	328 (38.1)	68 (32.1)	9 (28.1)	<0.001
Hypertension	1,687 (67.5)	871 (62.6)	624 (72.4)	163 (76.9)	28 (87.5)	<0.001
Diabetes mellitus	940 (37.6)	524 (37.7)	307 (35.6)	87 (41.0)	20 (62.5)	0.010
Dyslipidemia	1,872 (76.1)	1,050 (76.6)	642 (75.4)	150 (72.8)	28 (93.3)	0.219
Familial history of CAD	304 (12.2)	182 (13.1)	101 (11.7)	18 (8.5)	3 (9.4)	0.229
Previous MI	880 (35.2)	465 (33.4)	312 (36.2)	90 (42.5)	13 (40.6)	0.069
Heart failure	417 (16.7)	143 (10.3)	168 (19.5)	89 (42.0)	16 (50.0)	<0.001
LVEF	53.1 ± 8.6	54.4 ± 7.5	52.3 ± 9.1	49.2 ± 10.0	46.3 ± 10.7	<0.001
**Baseline medication**						
Aspirin	2,409 (96.4)	1,363 (98.0)	815 (94.5)	200 (94.3)	29 (90.6)	<0.001
Clopidogrel	2,321 (92.9)	1,295 (93.1)	801 (92.9)	192 (90.6)	31 (96.9)	0.479
Statin	2,381 (95.3)	1,337 (96.1)	813 (94.3)	199 (93.9)	30 (93.8)	0.172
β blocker	1,858 (74.3)	1,045 (75.1)	629 (73.0)	160 (75.5)	23 (71.9)	0.684
ACEI or ARB	1,567 (62.7)	851 (61.2)	560 (65.0)	137 (64.6)	17 (53.1)	0.173
One CTO lesion	2,140 (85.6)	1,211 (87.1)	725 (84.1)	172 (81.1)	30 (93.8)	0.031
Two CTO lesions	334 (13.4)	168 (12.1)	128 (14.8)	36 (17.0)	2 (6.3)	0.065
LAD	855 (34.2)	487 (35.0)	292 (33.9)	69 (32.5)	6 (18.8)	0.356
LCX	727 (29.1)	397 (28.5)	261 (30.3)	60 (28.3)	9 (28.1)	0.820
RCA	1,226 (49.1)	660 (47.4)	424 (49.2)	123 (58.0)	18 (56.3)	0.031
Multivessel disease	2,017 (80.8)	1,100 (79.2)	705 (81.8)	179 (84.4)	31 (96.9)	0.017
Proximal or mid CTO	1,770 (70.8)	972 (69.9)	621 (72.0)	153 (72.2)	24 (75.0)	0.673
Calcification	452 (18.1)	187 (13.4)	175 (20.3)	77 (36.3)	12 (37.5)	<0.001
Long lesions (≥20 mm)	1,624 (65.0)	881 (63.3)	567 (65.8)	150 (70.8)	25 (78.1)	0.064
J-CTO score	1.77 ± 1.19	1.71 ± 1.18	1.79 ± 1.18	2.00 ± 1.22	2.22 ± 1.29	0.002
SYNTAX score	22.6 ± 8.8	21.6 ± 9.4	24.0 ± 7.9	22.4 ± 8.5	21.7 ± 8.9	0.042

*Values are given as the mean ± standard deviation or numbers and percentages*.

*ACEI, angiotensin-converting enzyme inhibitor; ARB, angiotensin-receptor blocker; CAD, coronary artery disease; CTO, chronic total occlusion; eGFR, estimated glomerular filtration rate; J-CTO, Japanese-chronic total occlusion; LAD, left ascending coronary artery; LCX, left circumflex coronary artery; LVEF, left ventricular ejection fraction; MI, myocardial infarction; RCA, right coronary artery*.

**Table 2:** Procedural characteristics.

**Variables**	**CTO PCI (*n* = 1,220)**
Antegrade approach	1,107 (90.7)
Retrograde approach	113 (9.2)
IVUS use	74 (6.1)
OCT use	60 (4.9)
Number of stents	1.46 ± 0.75
Total stent length, mm	29.6 ± 24.0
Contrast volume, ml	226 ± 80
Coronary dissection	46 (3.8)
Coronary perforation	13 (1.1)
In-hospital death	12 (0.9)

*Values are given as the mean ± standard deviation or numbers and percentages*.

*CTO, chronic total occlusion; IVUS, intravenous ultrasound; OCT, optical coherence tomography; PCI, percutaneous coronary intervention*.

### Long-Term Outcomes

**Figure 1:**
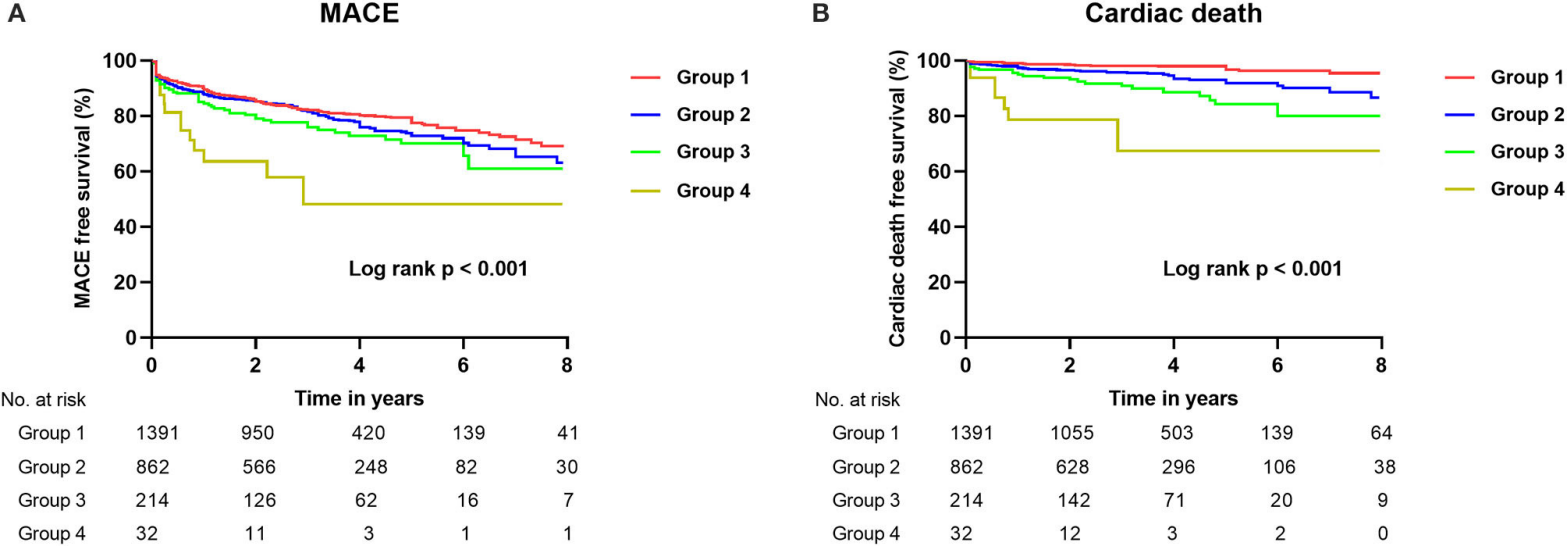
Kaplan–Meier curves for MACE **(A)** and cardiac death **(B)** during follow-up in total patients with CTO according to baseline renal function. CTO, chronic total occlusion; MACE, major adverse cardiovascular events; Group 1 [estimated glomerular filtration rate (eGFR) ≥ 90 ml/min/1.73 m^2^]; Group 2 (60 ≤ eGFR < 90 ml/min/1.73 m^2^); Group 3 (30 ≤ eGFR < 60 ml/min/1.73 m^2^); Group 4 (eGFR <30 ml/min/1.73 m^2^).

During a median follow-up of 2.6 (interquartile range, 1.2–4.7) years, MACE (*p* <0.001), cardiac death (*p* < 0.001), and MI (*p* < 0.001) were increased with decreasing renal function. However, the incidence of TVR (*p* < 0.001) was decreased with decreasing renal function (Table 3) (Figure 1).

**Table 3:** Clinical outcomes stratified according to baseline renal function.

**Variables**	**Group 1 (eGFR ≥ 90 ml/min/1.73 m^**2**^)**	**Group 2 (60 ≤ eGFR <90 ml/min/1.73 m^**2**^)**	**Group 3 (30 ≤ eGFR <60 ml/min/1.73 m^**2**^)**	**Group 4 (eGFR <30 ml/min/1.73 m^**2**^)**	***P* value**
	***n* = 1,391**	***n* = 862**	***n* = 212**	***n* = 32**	
MACE	265 (19.1)	186 (21.6)	54 (25.2)	13 (40.6)	<0.001
Cardiac death	31 (2.2)	53 (6.1)	24 (11.3)	7 (21.9)	<0.001
MI	79 (5.7)	73 (8.5)	26 (12.3)	5 (15.6)	<0.001
TVR	185 (13.3)	97 (11.3)	18 (8.5)	1 (3.1)	0.027

*Values are given as numbers and percentages*.

*eGFR, estimated glomerular filtration rate; MACE, major adverse cardiovascular events; MI, myocardial infarction; TVR, target-vessel revascularization*.

**Figure 2:**
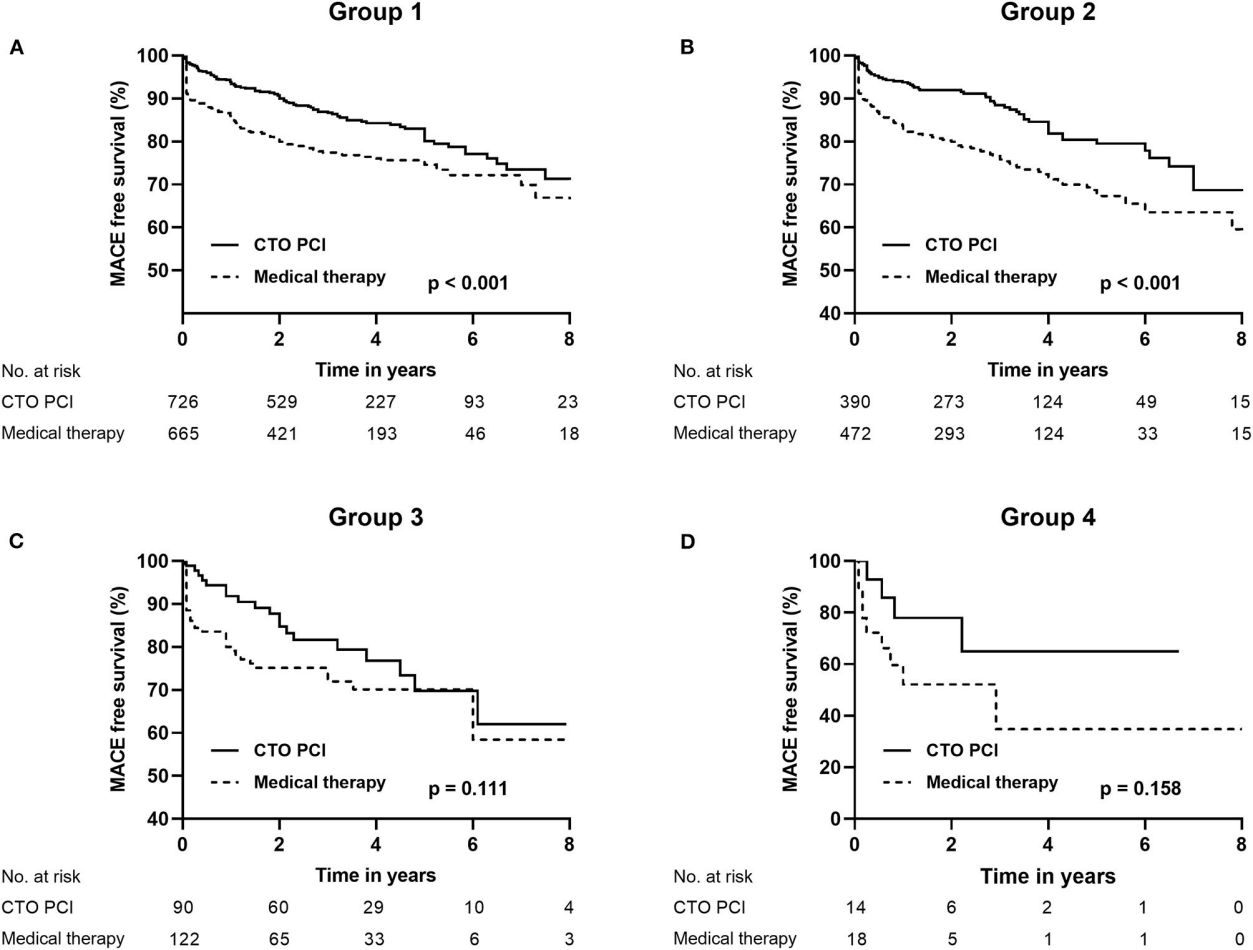
Kaplan–Meier curves for MACE **(A–D)** during follow-up for CTO PCI vs. medical therapy in CTO patients according to baseline renal function (groups 1, 2, 3, and 4). CTO, chronic total occlusion; MACE, major adverse cardiovascular events; PCI, percutaneous coronary intervention; Group 1 [estimated glomerular filtration rate (eGFR) ≥ 90 ml/min/1.73 m^2^]; Group 2 (60 ≤ eGFR < 90 ml/min/1.73 m^2^); Group 3 (30 ≤ eGFR < 60 ml/min/1.73 m^2^); Group 4 (eGFR <30 ml/min/1.73 m^2^).

The clinical outcomes of the two different strategies (MT and CTO PCI) for CTO patients in the four groups are shown in Table 4. In group 1 and group 2, MACE occurred less frequently in patients with CTO PCI, as compared to patients in the MT group (15.6% vs. 22.8%, *p* < 0.001; 15.6% vs. 26.5%, *p* < 0.001, respectively). However, there was no significant difference on MACE between CTO PCI and MT in group 3 (21.1% vs. 28.7%, *p* = 0.211) and group 4 (28.6% vs. 50.0%, *p* = 0.289). The differences were not significant on cardiac mortality and MI regarding the two treatment strategies among the four groups (Figure 2).

**Table 4:** Clinical outcomes of all patients stratified by eGFR in the medical therapy and PCI groups.

**Variables**	**Group 1**			**Group 2**			**Group 3**			**Group 4**		
	**MT**	**PCI**	***P* value**	**MT**	**PCI**	***P* value**	**MT**	**PCI**	***P* value**	**MT**	**PCI**	***P* value**
	***n* = 665**	***n* = 726**		***n* = 472**	***n* = 390**		***n* = 122**	***n* = 90**		***n* = 18**	***n* = 14**	
MACE	152 (22.8)	113 (15.6)	0.001	125 (26.5)	61 (15.6)	<0.001	35 (28.7)	19 (21.1)	0.211	9 (50.0)	4 (28.6)	0.289
Cardiac death	17 (2.5)	14 (1.9)	0.428	35 (7.4)	18 (4.6)	0.089	15 (12.3)	9 (10.0)	0.602	5 (27.8)	2 (14.3)	0.426
MI	41 (6.2)	38 (5.2)	0.453	47 (10.0)	26 (6.7)	0.084	13 (10.7)	13 (14.4)	0.406	3 (16.7)	2 (14.3)	0.999
TVR	107 (16.1)	78 (10.7)	0.011	65 (13.8)	32 (8.2)	0.010	13 (10.7)	5 (5.6)	0.188	0 (0)	1 (7.1)	0.438

*Values are given as numbers and percentages*.

*eGFR, estimated glomerular filtration rate; MACE, major adverse cardiovascular events; MI, myocardial infarction; MT, medical therapy; PCI, percutaneous coronary intervention; TVR, target-vessel revascularization*.

To reduce the impact of failed CTO procedures on outcomes, we compared the clinical outcomes of MT vs. successful CTO PCI in Table 5. In group 1 and group 2, rate of MACE was significantly reduced for patients who received successful CTO PCI compared to patients with MT (16.7% vs. 22.8%, *p* = 0.006; 16.3% vs. 26.5%, *p* = 0.003; respectively). However, we did not observe a significant difference on MACE between successful CTO PCI and MT in group 3 (23.3% vs. 28.7%, *p* = 0.492) and group 4 (37.5% vs. 50.0%, *p* = 0.683). There were no significant differences in terms of MI and TVR regarding the successful CTO PCI and MT among the four groups.

**Table 5:** Clinical outcomes of all patients stratified by eGFR in the medical therapy and successful PCI groups.

**Variables**	**Group 1**			**Group 2**			**Group 3**			**Group 4**		
	**MT**	**S-PCI**	***P* value**	**MT**	**S-PCI**	***P* value**	**MT**	**S-PCI**	***P* value**	**MT**	**S-PCI**	***P* value**
	***n* = 665**	***n* = 426**		***n* = 472**	***n* = 233**		***n* = 122**	***n* = 43**		***n* = 18**	***n* = 8**	
MACE	152 (22.8)	71 (16.7)	0.006	125 (26.5)	38 (16.3)	0.003	35 (28.7)	10 (23.3)	0.492	9 (50.0)	3 (37.5)	0.683
Cardiac death	17 (2.5)	10 (2.3)	0.764	35 (7.4)	8 (3.4)	0.038	15 (12.3)	4 (9.3)	0.597	5 (27.8)	1 (12.5)	0.628
MI	41 (6.2)	23 (5.4)	0.603	47 (10.0)	16 (6.9)	0.176	13 (10.7)	7 (16.3)	0.331	3 (16.7)	2 (25.0)	0.628
TVR	107 (16.1)	52 (12.2)	0.040	65 (13.8)	24 (10.3)	0.192	13 (10.7)	3 (7.0)	0.483	0 (0)	1 (12.5)	0.308

*Values are given as numbers and percentages*.

*eGFR, estimated glomerular filtration rate; MACE, major adverse cardiovascular events; MI, myocardial infarction; MT, medical therapy; PCI, percutaneous coronary intervention; S-PCI, successful PCI; TVR, target-vessel revascularization*.

After multivariate analysis, Cox model identified eGFR <30 ml/min/1.73 m^2^ [hazard ratio (HR) 2.53, 95% confidence interval (CI): 1.38–4.64, *p* = 0.003], MT (HR 1.59, 95% CI: 1.32–1.91, *p* < 0.001), age (per-year increment) (HR 1.01, 95% CI: 1.00–1.02, *p* = 0.005), male gender (HR 1.39, 95% CI: 1.11–1.76, *p* = 0.005), diabetes mellitus (HR 1.39, 95% CI: 1.16–1.67, *p* < 0.001), heart failure (HR 1.46, 95% CI: 1.16–1.84, *p* = 0.001), and multivessel disease (HR 1.57, 95% CI: 1.19–2.07, *p* = 0.001) as independent predictors for MACE in patients with CTOs. However, 30 ≤ eGFR < 60 ml/min/1.73 m^2^ (*p* = 0.433) and LVEF ≤ 40% (*p* = 0.690) were not associated with MACE in this cohort (Table 6).

**Table 6:** Univariate and multivariate analysis for MACE.

	**Univariate analysis**		**Multivariate analysis**	
**Variables**	**HR (95% CI)**	***P* value**	**HR (95% CI)**	***P* value**
eGFR ≥ 90 ml/min/1.73 m^2^	Reference		Reference	
60 ≤ eGFR <90 ml/min/1.73 m^2^	1.15 (0.95–1.39)	0.138	1.08 (0.87–1.34)	0.447
30 ≤ eGFR <60 ml/min/1.73 m^2^	1.43 (1.07–1.92)	0.016	1.15 (0.81–1.62)	0.433
eGFR <30 ml/min/1.73 m^2^	2.99 (1.71–5.23)	<0.001	2.53 (1.38–4.64)	**0.003**
From 2013 to 2018	0.89 (0.78–1.09)	0.736		
Medical therapy	1.73 (1.44–2.06)	<0.001	1.59 (1.32–1.91)	** <0.001**
Age (per-year increment)	1.01 (1.00–1.02)	0.001	1.01 (1.00–1.02)	**0.005**
Male	1.24 (1.00–1.55)	0.048	1.39 (1.11–1.76)	**0.005**
Smoking	1.12 (0.94–1.33)	0.208		
Hypertension	1.11 (0.93–1.34)	0.248		
Diabetes mellitus	1.39 (1.17–1.65)	<0.001	139 (1.16–1.67)	** <0.001**
Hyperlipidemia	0.91 (0.75–1.11)	0.354		
Previous MI	1.15 (0.97–1.37)	0.105		
Heart failure	1.70 (1.39–2.08)	<0.001	1.46 (1.16–1.84)	**0.001**
LVEF ≤ 40%	1.40 (1.11–1.76)	0.004	1.05 (0.81–1.36)	0.690
LAD CTO	0.95 (0.79–1.14)	0.624		
RCA CTO	1.23 (1.03–1.46)	0.020	1.19 (0.99–1.42)	0.055
Multivessel disease	1.80 (1.39–2.34)	<0.001	1.57 (1.19–2.07)	**0.001**

*CI, confidence interval; CTO, chronic total occlusion; eGFR, estimated glomerular filtration rate; HR, hazard ratio; LAD, left ascending coronary artery; LVEF, left ventricular ejection fraction; MACE, major adverse cardiovascular events; MI, myocardial infarction; RCA, right coronary artery. Bold values mean p < 0.05 and there is a statistical difference*.

## Discussion

This study demonstrated three major findings. Firstly, with the decline in renal function, patients tended to be older and had more comorbidities, including hypertension, diabetes mellitus, heart failure, and low LVEF. Secondly, MACE and cardiac death were increased with decreasing renal function. Thirdly, initial CTO PCI procedures were associated with reduced MACE in patients without CKD, but not in patients with CKD. Similar beneficial effects were observed in patients without CKD who underwent successful CTO procedures. To the best of our knowledge, this is the first large cohort study to report the impact of renal function on long-term clinical outcomes in patients with coronary CTOs.

Renal impairment is associated with inflammatory, oxidative stress, and metabolic perturbations and coronary microcirculation disorders (1, 19–22), all of which are associated with accelerated atherosclerosis and endothelial dysfunction and increased incidence of hypertension, diabetes mellitus, dyslipidemia, multivessel disease, and coronary calcified lesions (23, 24). These may be the mechanisms that renal dysfunction contribute to adverse short-term as well as long-term outcomes. In the previous studies, a cardiologist evaluated the outcomes after revascularization in CAD patients with renal impairment, and they found that the presence of renal impairment was associated with lower success rate and increasing complication and cardiac death or other adverse cardiovascular events (3, 25, 26).

CTO remains a challenging obstacle in coronary intervention (27). The benefits from successful revascularization for CTO, such as reduced death and angina, had been proved by several studies (9, 28). The large registry reported that PCI for CTO lesions account for <5% of coronary interventions in clinical practice (29), and most of the patients with CTO lesions still are treated conservatively (10, 30). A prior study reported that the prevalence of CTO in a non-infarct-related artery was 13% in patients with renal insufficiency compared with 7% in those with normal renal function (10). The number of CTO patients with renal insufficiency was huge. Previous studies have mainly focused on the impact of renal insufficiency on clinical outcomes after revascularization among acute coronary syndrome patients or unselected patients. However, patients with significant renal insufficiency are regularly excluded from most studies relevant to CTO, and little data exist on the impact of renal impairment on outcomes in among patients with CTOs. Furthermore, which strategy is optimal in this high-risk population remains unknown. Consequently, evaluating clinical outcomes of CTO PCI vs. MT is crucial for decision making in clinical practice among patients with renal dysfunction.

Our study provides evidence that, with decreasing renal function, the prevalence of MACE and cardiac death was increased, in accordance with the findings of Stähli and coworkers (31). Furthermore, we found that PCI for CTO was associated with reduced MACE compared to MT alone among patients without CKD. Nonetheless, no beneficial effects of CTO PCI were observed in patients with 30 ≤ eGFR <60 ml/min/1.73 m^2^ and patients with eGFR <30 ml/min/1.73 m^2^. These findings were supported by recent studies, which showed no decreased adverse outcomes following CTO PCI in patients with CKD (32, 33). Of note, even in the recent ISCHEMIA-CKD trial, revascularization for stable CAD did not improve outcomes among patients with advanced CKD (eGFR <30 ml/min/1.73 m^2^), as compared with medical treatment (34). Additionally, we found that eGFR <30 ml/min/1.73 m^2^ was a strong predictor of MACE in CTO patients, which were consistent with findings of previous studies (9, 31).

Of note, failure of CTO PCI was associated with increasing baseline patient and lesion complexity, and therefore, the poorer clinical outcomes were more common among this population (8, 13). To reduce the impact of failed CTO procedures on outcomes, we compared the clinical outcomes of MT vs. successful CTO PCI. We found that successful CTO PCI was associated with reduced MACE compared to MT alone among patients without CKD but not in patients with CKD, which was similar with the results of MT vs. initial CTO PCI.

Up to now, no guideline or widely recognized consensus on treatment strategy of CTO patients with renal insufficiency and the prognosis of this population is unknown. Therefore, our findings strengthen the idea that a careful patient selection is important and suggest that, considering prognosis as well as the multiple comorbidities, operative complications, and high expense, aggressive revascularization should be considered carefully for these high-risk patients with significant renal insufficiency.

Limitations should be taken into consideration. Firstly, the analysis is limited by an observational design and the patients with CKD is relatively small. Secondly, as the time period of the study is long (more than 10 years), the changes of PCI materials and technique or medical treatment may have the impact on the clinical outcomes, though we have considered these factors in our previous study (4). Additionally, the infrequent use of imaging devices, such as IVUS and OCT, due to high cost in the present study may lower the success rate of CTO PCI and affect the outcomes, though our success rate was similar to the National Cardiovascular Data Registry from the US (29). Thirdly, the crossovers of MT and CTO PCI groups may limit conclusions, even though the rate was very low. Despite these limitations, this is the first large cohort study to report different treatment strategies on clinical outcomes in patients with coronary chronic total occlusions according to baseline renal function, and our study provided valuable data among this high-risk population, which may benefit future investigations.

## Conclusions

With decreasing renal function, the prevalence of MACE and cardiac death was increased among patients with CTOs. For treatment of CTO, compared with MT alone, CTO PCI may reduce the risk of MACE in patients without CKD. However, reduced MACE from CTO procedures among patients with CKD was not observed. Similar beneficial effects were observed in patients without CKD who underwent successful CTO procedures. Large randomized clinical trials are required to confirm the findings of the present study.

## Data Availability Statement

The datasets generated for this study are available on motivated request to the corresponding author.

## Ethics Statement

The studies involving human participants were reviewed and approved by First Affiliated Hospital of Dalian Medical University. The patients/participants provided their written informed consent to participate in this study.

## Author Contributions

LG and HD prepared the manuscript. All authors contributed to data collection and analyses, edited the draft manuscript, and approved the final manuscript.

## Conflict of Interest

The authors declare that the research was conducted in the absence of any commercial or financial relationships that could be construed as a potential conflict of interest.
