# Corrigendum: Impact of Renal Function on Long-Term Clinical Outcomes in Patients With Coronary Chronic Total Occlusions: Results From an Observational Single-Center Cohort Study During the Last 12 Years

**DOI:** 10.3389/fcvm.2020.638164

**Published:** 2021-01-08

**Authors:** Lei Guo, Huaiyu Ding, Haichen Lv, Xiaoyan Zhang, Lei Zhong, Jian Wu, Jiaying Xu, Xuchen Zhou, Rongchong Huang

**Affiliations:** ^1^Department of Cardiology, The First Affiliated Hospital of Dalian Medical University, Dalian, China; ^2^Department of Radiology, Fuyang Hospital of Anhui Medical University, Fuyang, China; ^3^Department of Cardiology, Capital Medical University Affiliated Beijing Friendship Hospital, Beijing, China

**Keywords:** chronic total occlusions, medical therapy, outcomes, percutaneous coronary intervention, renal function

In the published article, there was an error in affiliation **1**. Instead of “**Rongchong Huang**^**3**^,” it should be “**Rongchong Huang**^**1,3**^.”

The authors apologize for this error and state that this does not change the scientific conclusions of the article in any way. The original article has been updated.

